# Randomized controlled trial of Hepatitis B virus vaccine in HIV-1-infected patients comparing two different doses

**DOI:** 10.1186/1742-6405-3-9

**Published:** 2006-04-06

**Authors:** Patricia Cornejo-Juárez, Patricia Volkow-Fernández, Kenia Escobedo-López, Diana Vilar-Compte, Guillermo Ruiz-Palacios, Luis Enrique Soto-Ramírez

**Affiliations:** 1 Infectious Diseases, Instituto Nacional de Cancerología, Mexico City, México. Av. San Fernando No. 22, Col. Sección XVI, Tlalpan, 14000 México, D.F, Mexico; 2 Infectious Diseases, Instituto Nacional de Ciencias Médicas y de la Nutrición. Salvador Zubirán, Mexico City, Mexico

## Abstract

**Background:**

Co-infection with hepatitis B virus (HBV) and human immunodeficiency virus (HIV) is not infrequent as both share same route of exposure. The risk of developing chronic hepatitis B virus is 6%, in general population but can reach 10–20% in HBV/HIV co-infected patients. When compared to general population, the response rate to HBV vaccine in HIV-infected patients is diminished, so previous studies have tried to improve this response using variety of schedules, doses and co-administration of immunomodulators. The purpose of this study was to evaluate two doses of recombinant HBV vaccine (10 or 40 μg), IM at 0, 1 and 6 months. Vaccination response was measured 30–50 days after last dose; titers of >9.9 IU/L were considered positive.

**Results:**

Seventy-nine patients were included, 48 patients (60.7%) serconverted. Thirty-nine patients (49.3%) received 10 μg vaccine dose, 24 patients (61.5%) seroconverted. Forty patients (50.7%) received 40 μg vaccine dose, 24 (60%) seroconverted. There were no differences between two doses. A statistically significant higher seroconversion rate was found for patients with CD4 cell counts at vaccination ≥ 200 cel/mm3 (33 of 38 patients, 86.8%), compared with those with CD4 < 200 cel/mm3 (15 of 41, 36.6%), [OR 11.44, 95% IC 3.67–35.59, p = 0.003], there were no differences between two vaccine doses. Using the logistic regression model, CD_4 _count <200 cel/mm^3 ^were significantly associated with non serologic response (p = 0.003). None other variables such as gender, age, risk exposure for HIV, viral load, type or duration of HAART or AIDS-defining illness, were asociated with seroconversion.

**Conclusion:**

In this study, an increase dose of HBV vaccine did not show to increase the rate of response in HIV infected subjects. The only significant findings associated to the response rate was that a CD4 count ≥ 200 cel/mm^3^, we suggest this threshold at which HIV patients should be vaccinated.

## Background

Hepatitis B virus (HBV) is one of the major causes of acute and chronic hepatitis worldwide that can be prevented by immunization [1,2].

Co-infection with HVB and human immunodeficiency virus (HIV) is frequent as both share the same routes of transmission [3]. In general population, risk of developing chronic hepatitis is 6%, but it can reach 10–20% in HBV/HIV co-infected patients, besides this HBV/ HIV patients present a higher level of HBV replication and potential of transmission is increased [2,4-8]. HBV infection has been associated with more rapid progression to AIDS, explained by an increased expression of HIV-infected cells and faster decrease in CD4 lymphocytes [9-12].

When compared to general population, the response rate to HBV vaccine in HIV-infected patients, is diminished (40–60% vs 60–80%) [10,13]. This lower response is related with CD4 count less than 500 cel/mm3, and has also been found with other antigens like influenza or pneumococcal vaccines [14,15].

In previous studies including patients under hemodialysis, the rate of response to HBV vaccine has been significantly augmented by increasing dose, giving a fourth dose of the vaccine or using immunomodulators agents such as levamisole [2,16]. In HIV-patients the use of granulocyte-macrophage colony-stimulating factor (GM-CSF) concurrent with HBV vaccine, has shown a significant increase in seroconversion rate and in anti-HBs titers [17].

Currently, there are no data to determine the best HBV vaccine schedule for HIV-infected patients. With the aim to evaluate the rate of response to two different concentration of HBV vaccine in HIV-infected patients, we conducted a controlled, randomized, clinical trial. We also evaluated HIV viral load and CD_4 _counts at the time of vaccination.

## Methods

We conducted a double blind, randomized, controlled trial using two different concentrations of HBV vaccine 10 or 40 μgs (*Recombivax*, *HB*, *Merck*, *Sharp *&*Dohme*, *USA*), in two groups of HIV-infected patients stratified by CD4 count at time of vaccination (< 200 or ≥ 200 cel/mm^3^) attending an HIV/AIDS Clinic at the Instituto Nacional de Ciencias Médicas y de la Nutrición Salvador Zubirán and at the Instituto Nacional de Cancerología in Mexico City. The study was reviewed and approved by the Institutional Committee of Human Biomedical Investigation (CIBH: 860 and CFEI: INF-0599900-1, approved on December 1999).

We included HIV-infected patients >16 years of age, negative for any HBV serological marker, not previously vaccinated, without active opportunistic infection at the time of vaccination, who accepted to participate and signed informed consent.

Patients were randomized to receive 10 or 40 μg of HBV recombinant vaccine, 1 ml intramuscularly in the deltoid region at 0, 1 and 6 months. We collected data on age, gender, route to exposure for HIV infection, date for HIV infection diagnosis, CD4 count and HIV viral load at the first vaccine dose, type and time (in months) under antiretroviral treatment and AIDS-defining event.

A technician who ignored vaccine dose, administered the vaccine and collected a serum sample 40 ± 10 days after third-dose application.

Quantitative anti-HBs test by IU/L (Microelisa system, Hepanostika^® ^Anti-HBs New, Organon Tecknika, The Netherlands) was performed. Negative samples for quality assurance were included. All sera were tested simultaneously. Response to vaccination was considered when there was a rise in anti-HBs titers ≥ 10 IU/L. The absolute count of CD4 lymphocytes was determined by a fuorescence-activate cell analyzer, using monoclonal antibodies. The quantization of HIV-1 RNA was measured by AMPLICOR HIV-1 MONITOR^® ^test was from 40 to 750,000 RNA copies/mL.

### Statistical analysis

We calculated an estimated 60% seroconversion rate for the standard dose, an increase of 20% for the double dose to be clinically significant. Eighty patients in each group was required for a clinically difference.

We calculated seroconversion rate for each vaccine dose by mean ± standard deviation for Student *t *test or Mann-Whitney test for continuous variables were used as appropriate. For discrete variables, we used Chi-square or Fisher exact test and reported odds ratios (ORs) with 95% confidence interval (95% CI). P values ≤ 0.05 were considered statistically significant.

Univariate analysis was used to test for associations between independent (age, gender, vaccine dose, CD4 count and viral load at time of vaccination, time in months from HIV diagnosis, treatment with HAART and AIDS-defining event) and dependent variable (seroconversion). A logistic regression model and a Cox model were performed.

## Results

Patients were recruited between April 1999 and May 2000. Eighty four patients were included. Five (6%) were lost during follow-up [two (2.4%) in 10 μg dose and three (3.6%) in 40 μg dose]. Characteristics of subjects who completed the study and those who dropped out were similar.

Non significant differences were found among demographic variables between the two groups. Age, gender, CD4 count at vaccination, HIV viral load, history of an AIDS defining event and antiretroviral therapy for each group are depicted in Table 1.

**Table 1:** Characteristics of HIV-infected patients. Baseline clinical and demographic characteristics of HIV-infected patients, who completed the study (n = 79)

**Variables**	**Vaccine 10 μg**	**Vaccine 40 μg**	**p**
No. patients (%)	39 (49.3)	40 (50.7)	-

Gender male – No. (%)	27 (69.2)	29 (72.5)	0.749

Mean age (years ± s.d.)	35.6 ± 8.12	34.1 ± 7.6	0.378

HIV exposure – No. (%)			
Heterosexual	18 (45%)	16 (41%)	0.721
Homo or bisexual	22 (55%)	23 (59%)	

Diagnosis of HIV (months ± s.d.)	40.6 ± 35.4	40.2 ± 32.65	0.960

Mean CD4 count (cel/mm^3 ^± s.d.)	245 ± 217.9	225.45 ± 189.7	0.671

CD4 cel/mm^3 ^/ No. (%)			
< 200	20 (51.3%)	21 (52.5%)	0.914
≥ 200	19 (48.8%)	19 (47.5%)	

Viral load (copies/mL)	75,187 ± 153,305	67,335 ± 112,742	0.811

Viral load (copies/mL) – No. (%)			
≤ 400	6 (20%)	9 (25%)	0.860
400 – ≤ 20,000	11 (36.6%)	11 (30.6%)	
≥ 20,000	13 (43.3%)	16 (44.4%)	

AIDS-defining illness – No. (%)	17 (43.6)	10 (25)	0.082

Treatment with HAART* – No. (%)	22 (56.4)	29 (72.5)	0.135
PI	20 (51.2)	23 (57.5)	
NNRTI	1 (2.6)	4 (10)	
PI + NNRTI or 3 NRTI	1 (2.6)	2 (5)	

PI: Protease inhibitor; NNRTI: non-nucleoside reverse transcriptase inhibitor; NRTI: Nucleoside reverse transcriptase inhibitor.

The overall seroconversion rate after HBV vaccination was 60.7% (48 of 79 patients). For 10 μg vaccine dose, 24 of 39 patients (61.5%) seroconvert; and for 40 μg vaccine dose, 24 of 40 patients (60%). Non significant difference was found between two different vaccine concentrations [relative risk (RR) = 1.1; 95% confidence interval (CI) = 0.61–1.98, p = 0.889].

Stratified by CD4 count, 33 of 38 patients (86.8%) with CD4 ≥ 200 cel/mm3 seroconverted, compared with 15 of 41 patients with < 200 cel/mm3 (36.5%), (OR = 11.4, 95% CI = 3.6–35.6, p = 0.003). Stratified by viral load, 12 of 15 patients with < 400 copies/mL seroconverted (80%), and 30 of 51 patients with ≥ 400 copies/mL (58.8%), (OR 0.45, 95% CI= 0.22–0.92, p = 0.29).

Patients with CD4 < 200 cel/mm3 and viral load < 400 copies/mL, showed higher seroconversion rates, but only CD4 count was statistically significant. No diference was observed with two different vaccine doses.

Variables included in the logistic regression model were vaccine dose, CD4 count, viral load, HAART treatment, AIDS-defining illness, gender and risk factor for HIV infection. Only CD4 count <200 cel/mm3 was associated with non seroconversion.

**Figure 1:**
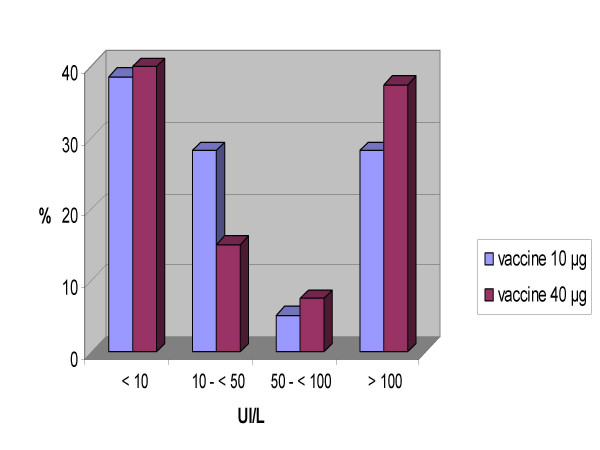
**Titers post-vaccination**. Titers post-vaccination categorized in four groups, with two different vaccine concentrations (UI/mL).

Mean anti-HBs titers were 137.3 ± 56.7IU/L for the 10 μg vaccine dose and 144.1 IU/L ± 56.7 for the 40 μg vaccine dose (p=ns). Titers post-vaccination are shown in Figure 1. Titers were significantly higher in patients with CD4 ≥ 200 cel/mm^3 ^compared with those with CD4 < 200 cel/mm^3 ^(107.2 ± 56.7 IU/L vs 39.7 ± 35.4 IU/L, p < 0.005).

HBV vaccine was well tolerated by all patients; two patients reported pain at the injection site, one with erythema. No serious adverse events were registered.

## Discussion

Approximately 90–97% of healthy adults will show protective anti-HBs titers after vaccination with recombinant HBV vaccine [18,19]. As previously reported [7,20,21], we found a lower rate of response in this cohort of HIV-infected patients vaccinated with HBV recombinant vaccine (60.7%) of the population fully immunized, increasing vaccine did not have a beneficial effect.

Risk factors significantly associated to failure of vaccination in previously reports, were the degree of immunosuppression and clinical markers of advanced HIV disease like CD4 count at vaccination and history of an AIDS-defining event. We found that the factor most strongly associated with non seroconversion and lower anti-HBs titers was CD_4 _count <200 cel/mm^3^. Previous studies with other antigens (like influenza or 23-valent pneumococcal vaccines) have shown lesser response asocciated with lower CD4 counts [14,15].

There are numerous reports describing a variety of dose schedules, limited success and markers associated with impaired response to HBV vaccine in these individuals. Most studies have been small in size sample making it difficult to draw conclusions within and between studies. Recently Fonseca et al, found higher serconversion with double vaccine dose in those patients with CD4 count ≥ 350 cel/mm^3 ^and low HIV viremia, with no differences between two different vaccine doses in patients with CD4 < 350 cel/mm^3 ^[22].

This study was performed with a smaller sample that initially calculated as we found trouble in getting non vaccinated or non infected HBV patients. Because of the small size, the power to determine differences between the two dosages of vaccine is low resulting in the possibility of a type II error. We found that the CD4 nadir (<200 cel/mm^3^) of patients whose CD_4 _increase with HAART over ≥ 200 had a similar rate of response when compared to patients with persistent CD_4_≥ 200 cel/mm^3^(data not presented); this finding should be interpreted with caution, it could be related to the small sample size. One interesting point is to investigate in the future the duration of this increase to achieve best rate of response in HIV-infected patients receiving HAART.

No seroconversion differences were found between this risk groups among homosexual, bisexual or heterosexual patients as has previously reported in other studies [1,2,18,20]. In this sample no patients had a history of drug abuse probably to the low number of HIV infected patients associated to this risk factor in Mexico less than 1%. None other risk factors as age, gender, type or duration of HAART or history of AIDS-defining event were related with serconversion.

We did not find any serious adverse event related with HBV vaccination in this group of patients as HIV and HBV share the same routes of exposure, we recommend vaccinating HIV patients against HBV.

## Conclusion

Although the sample study is small to give a definite conclusion, increasing the does not appears to contribute to HVB vaccine seroconversion. This study confirms previous reports that HIV-infected patients have a poor immunologic response to HBV vaccine, but a CD4 count threshold is ≥ 200 cell/mm^3 ^appears to increase vaccination response independently to vaccine dosing, as has been show by other studies.

## Competing interests

The author(s) declare that they have no competing interests.

## Authors' contributions

PCJ- Participated in the design of the study, collected data, wrote the manuscript

PVF- Revising the manuscript, statistical analysis

KEL- Carried out immunoassays

DVC- Statistical analysis and revising the manuscript

GRP- Revising the manuscript critically for important intellectual content

LESR- Analysis and interpretation of data, revising the manuscript critically for important intellectual content

All authors read and approved the final manuscript.

**Table 2:** Logistic regression model. Independent variables associated with non-seroconversion

	OR*	**OR adjusted [CI **_95%]_	**p**
Vaccine dose 10 μg	-	0.937 ± 0.432 [0.27–2,31]	0.889
CD4 < 200 cel/mm3	1.21 [0.3–4.9]	11.44 ± 6.62 [3.67–35.59]	0.003
Viral load ≥ 40 copies/mL	0.82 [0.17–3.82]	0.451 ± 0.164 [0.22–0.92]	0.029
Non HAART treatment	0.58 [0.15–2.23]	0.649 ± 0.201 [0.35–1.19]	0.164
AIDS-defining illness	2.5 [0.54–12.29]	0.972 ± 0.333 [0.49–1.9]	0.934
Gender male	0.75 [0.13–4.27]	0.806 ± 0.266 [0.42–1.54]	0.517
Homosexual or bisexual	0.6 [0.16–2.2]	0.844 ± 0.251 [0.47–1.51]	0.569

* OR=odds ratio
